# Structural Characterization of *Clostridium sordellii* Spores of Diverse Human, Animal, and Environmental Origin and Comparison to *Clostridium difficile* Spores

**DOI:** 10.1128/mSphere.00343-17

**Published:** 2017-10-04

**Authors:** Rebecca Rabi, Lynne Turnbull, Cynthia B. Whitchurch, Milena Awad, Dena Lyras

**Affiliations:** a Infection and Immunity Program, Monash Biomedicine Discovery Institute and Department of Microbiology, Monash University, Clayton, Victoria, Australia; b ithree institute, University of Technology Sydney, Ultimo, NSW, Australia; University of Iowa

**Keywords:** *Clostridium*, *Clostridium difficile*, *Clostridium sordellii*, endospores, exosporium

## Abstract

*Clostridium sordellii* is an often-lethal bacterium causing human and animal disease. Crucial to the infectious cycle of *C. sordellii* is its ability to produce spores, which can germinate into toxin-producing vegetative bacteria under favorable conditions. However, structural details of the *C. sordellii* spore are lacking. Here, we used a range of electron microscopy techniques together with superresolution optical microscopy to characterize the *C. sordellii* spore morphology with an emphasis on the exosporium. The *C. sordellii* spore is made up of multiple layers with the exosporium presenting as a smooth balloon-like structure that is open at the spore poles. Focusing on the outer spore layers, we compared the morphologies of *C. sordellii* spores derived from different strains and determined that there is some variation between the spores, most notably with spores of some strains having tubular appendages. Since *Clostridium difficile* is a close relative of *C. sordellii*, their spores were compared by electron microscopy and their exosporia were found to be distinctly different from each other. This study therefore provides new structural details of the *C. sordellii* spore and offers insights into the physical structure of the exosporium across clostridial species.

**IMPORTANCE** *Clostridium sordellii* is a significant pathogen with mortality rates approaching 100%. It is the bacterial spore that is critical in initiating infection and disease. An understanding of spore structures as well as spore morphology across a range of strains may lead to a better understanding of *C. sordellii* infection and disease. However, the structural characteristics of the *C. sordellii* spores are limited. In this work, we have addressed this lack of detail and characterized the *C. sordellii* spore morphology. The use of traditional and advanced microscopy techniques has provided detailed new observations of *C. sordellii* spore structural features, which serve as a reference point for structural studies of spores from other bacterial species.

## Abstract (precis)

*Clostridium sordellii* is a significant pathogen with mortality rates approaching 100%. It is the bacterial spore that is critical in initiating infection and disease. An understanding of spore structures as well as spore morphology across a range of strains may lead to a better understanding of *C. sordellii* infection and disease. However, the structural characteristics of the *C. sordellii* spores are limited. In this work, we have addressed this lack of detail and characterized the *C. sordellii* spore morphology. The use of traditional and advanced microscopy techniques has provided detailed new observations of *C. sordellii* spore structural features, which serve as a reference point for structural studies of spores from other bacterial species.

## INTRODUCTION

*Clostridium sordellii*, also termed *Paeniclostridium sordellii* (1), is a highly pathogenic Gram-positive, spore-forming anaerobe, infecting both humans and animals. *C. sordellii* infection in animals can result in enteritis (2–5), omphalitis (6), and equine atypical myopathy (7). In humans, a range of *C. sordellii* diseases have been recorded, the most common of which include toxic shock, bacteremia, and soft tissue infections (8). *C. sordellii* disease is toxin mediated, with up to 8 different toxins currently known to be produced by this bacterium (8). Distinct *C. sordellii* isolates can produce a different complement of toxins (9, 10). The major toxins are the glucosyltransferases TcsL and TcsH, which inactivate Rho GTPases, leading to cytoskeletal cellular disorganization (8). TcsL and TcsH are orthologs of the *Clostridium difficile* (also known as *Clostridioides difficile* [1]) TcdB and TcdA toxins, respectively, and are believed to be important for *C. sordellii*-mediated disease pathogenesis (11). Treatment regimens for *C. sordellii* infections are not well established or standardized; however, they include antibacterial therapy, wound debridement, and amputation. Despite treatment, the majority of *C. sordellii* infections result in death (8, 12), most likely as a consequence of toxin-induced damage that occurs in the host even once *C. sordellii* cells have been disabled (13).

Important to the infectious cycle of *C. sordellii* is its ability to produce spores. Spores are extremely hardy and able to persist under adverse conditions and in otherwise unsuitable environments for up to hundreds of years (14). *C. sordellii* spores are believed to be ubiquitous in nature, being present in the soil (15) and the gastrointestinal tracts of animals (2, 5) and humans (16), and are also found in the human vagina (16). Disease can be initiated when spores gain access to favorable niches through environmental contamination or self-contamination (8). Favorable conditions trigger spore germination, which results in vegetative cell formation. The vegetative cell then produces toxins that cause disease symptoms. In the case of soft tissue infections in injecting black tar heroin users, spore-contaminated soil is believed to be the source of *C. sordellii*. Skin-popping and speed-balling in these users lead to an increase in anaerobic conditions, which are favorable for spore germination (12).

Despite the importance of *C. sordellii* spores in initiating disease, they have not been studied recently, nor have they been studied in detail. Although previous studies have provided some morphological insights, the *C. sordellii* strains assessed have been limited in number and type with respect to host and geographic origin (17, 18). Papers published over 40 years ago show that the *C. sordellii* spore consists of a central region, which has been termed the inner spore in previous literature (19), surrounded by a loose balloon-like exosporium, with spores of several strains containing tubular appendages (17, 18). The exosporium is believed to be functionally important as it is the point of contact between the spore and the environment.

All *C. sordellii* spores studied to date have an exosporium (17, 18). The role of the exosporium in *C. sordellii* is unknown, and its role in the closely related bacterium *C. difficile* (20, 21) or in the more distantly related *Bacillus anthracis*, *Bacillus cereus*, and *Bacillus thuringiensis* (19, 22–25) is also not well understood. Some studies propose that the exosporium is involved in protecting the spore from degradative enzymes, antibodies, and macrophages, while others suggest that the exosporium helps to regulate spore germination and adherence to abiotic and biotic surfaces (19–25). Furthermore, the functional role of the tubular appendages in *C. sordellii* spores is unknown, although in *B. cereus* they are thought to be involved in spore adherence to abiotic surfaces (26). In addition, the structures of the *C. sordellii* inner spore have not been examined in any detail. Spores characterized from other clostridial species, and also *Bacillus* members, have an inner spore that consists of multiple structures. The innermost compartment of the spore, referred to as the core, contains a well-protected copy of the bacterial DNA. The core is surrounded by multiple layers that include the inner membrane (IM), germ cell wall (GCW), cortex (Cx), outer membrane, and the spore coat. In species such as *Bacillus subtilis* and *B. cereus*, the coat may be composed of three layers: an amorphic undercoat (UC) which surrounds the outer membrane, a laminated or striated inner coat (IC), and an electron-dense outer coat (OC). These inner spore structures are likely to play a role in spore germination and protection of the spore (14, 27–31). Given that there has been no published work on the *C. sordellii* inner spore structures to date, we have used electron microscopy in this study to identify these structures.

Previous studies characterizing *Clostridium* and *Bacillus* species spore morphology have typically visualized whole spores and spore sections by transmission electron microscopy (TEM). Few studies have used scanning electron microscopy (SEM), despite its ability to provide structural details of the spore surface. In addition, cryo-electron microscopy (cryo-EM) and superresolution optical microscopy have rarely been used in the study of spore morphology despite the advantage that these techniques offer in minimal sample preparation and enabling visualization of a hydrated spore. In this study, we used a whole-mount TEM method to visualize *C. sordellii* spores from 16 different strains chosen to represent a range of geographic locations, host origins, and each of the 4 clades into which *C. sordellii* has been classified based on core genome sequence similarities (9). In addition, we used a range of traditional and more advanced microscopy techniques to study spores isolated from the strain ATCC 9714, which is the *C. sordellii* type strain and is able to be genetically manipulated (32). Studying spores from a range of strains as well as using a range of microscopy techniques has provided novel insights into the morphology of *C. sordellii* spores.

Despite the structural and functional similarity between the toxins produced by *C. sordellii* (TcsL and TcsH) and *C. difficile* (TcdB and TcdA), infection with these bacteria results in very different diseases, with the latter causing antibiotic-associated diarrheal syndromes (11, 27). This difference in disease-causing capacity is likely to result from the different body niches that these two clostridial species can colonize and occupy, which may be defined by the spores that initiate these infections and the germination conditions that they encounter in these niches. Although data in previous studies suggest that the spores from *C. sordellii* (17, 18) are morphologically distinct from those of *C. difficile* (33), a direct and detailed comparison has not been performed. In this study, a comparison of *C. sordellii* and *C. difficile* spores, using some of the microscopy techniques described above, has shown that the exosporial structures between these spores are vastly different.

## RESULTS

### Morphology of spores isolated from diverse *C. sordellii* isolates.

**FIG 1:**
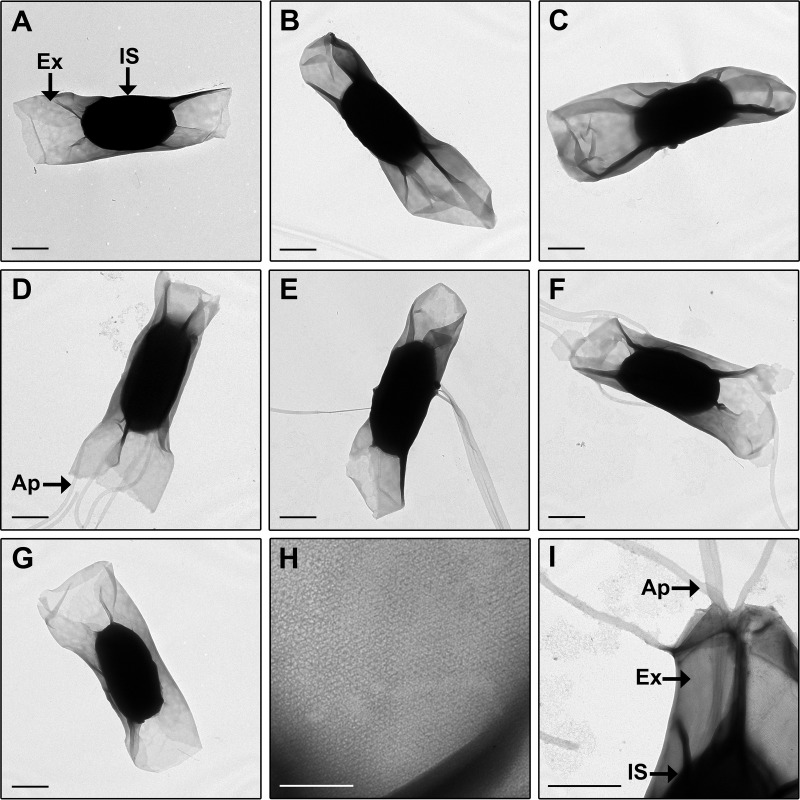
(A to G) TEM on negatively stained whole spores of *C. sordellii* strains ATCC 9714 (A), DA108 (B), JGS6382 (C), SSCC26591 (D), R28058 (E), W2922 (F), and W3026 (G). (H) Magnified view of exosporium showing hexagonal structural array. (I) Magnified view of spore appendages emerging through the exosporium for strain SSCC26591. Spores of all strains have a baggy exosporium (A to G); spores of some strains have tubular appendages (D to F). Ap, appendage; Ex, exosporium; IS, inner spore. Bars, 0.5 µm (A to G and I) and 0.1 µm (H).

The spore morphology of *C. sordellii* strains that represent clinical and animal isolates derived from different geographical locations and which also represent a range of clades was characterized by TEM imaging of the whole spore (Fig. 1). All spores had an electron-dense inner spore and a more electron-lucent exosporium, which appears as a loose baggy structure surrounding the inner spore. The exosporium in all strains was found to be located close to the inner spore at the longitudinal edge but was distantly located from the inner spore at the poles (Fig. 1A to G). Close examination of the exosporium revealed a hexagonally arranged ultrastructure (Fig. 1H).

**FIG 2:**
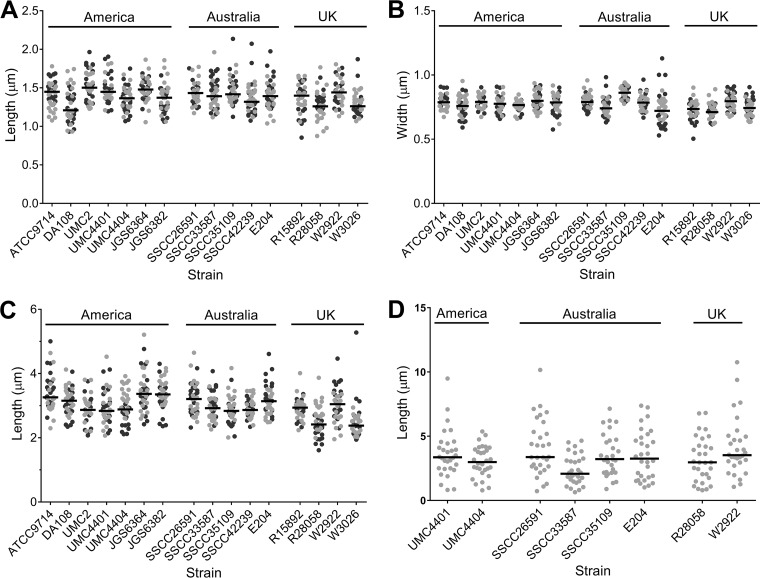
*C. sordellii* inner spore length (A), inner spore width (B), exosporial length (C), and appendage length (D). Values recorded for each spore measurement are plotted. Dark and light gray dots indicate different biological samples, with 20 spores from two biological samples each measured for inner spore length and width and exosporial length and 30 appendages measured from one biological sample for appendage length. Horizontal black lines indicate the median values. Strains are grouped according to country of origin. All strains are human isolates except for strains JGS6364, JGS6382, W2922, and W3026, which are animal isolates.

To determine if there were size differences in the inner spore and exosporium within or between the strains examined, measurements of these structures were performed (Fig. 2). Within each *C. sordellii* strain, there was a wide distribution in spore measurements, with spores showing the greatest variation in their exosporial lengths (Fig. 2C) and the least variation in their inner spore width (Fig. 2B). The greatest difference between strains was observed in the median exosporial length, with an almost 1-µm difference recorded between strains JGS6364 and W3026, which had the longest and shortest exosporia, respectively (Fig. 2C). Differences in inner spore measurements between strains were less pronounced than exosporial lengths, with a 0.29-µm difference in length noted between the longest and shortest median inner spore in strains UMC2 and DA108, respectively (Fig. 2A). The median inner spore width showed the least variation between strains, with only a 0.15-µm difference noted between the widest inner spore, which was found in strain SSCC35109, and the shortest inner spore, found in strain R28058 (Fig. 2B). Overall, there was very little variation in inner spore and exosporial size between the diverse strains under investigation in this study.

Tubular appendages were present on spores of 9 out of the 16 strains examined (Fig. 1D to F and I and 2D). The number of appendages per spore varied between strains, with between 1 and 4 seen on spores from strains UMC4404 and SSCC26591, 1 to 3 on SSCC33587 and W2922, 1 to 5 on UMC4401, 2 to 8 on SSCC35109, 1 to 7 on E204, and 1 to 8 on R28058. Within each strain, there was a wide variation in the appendage length recorded, and between strains, the median appendage length varied, ranging from 2.08 µm to 3.52 µm for strains SSCC33587 and W2922, respectively. Spores of 4 strains with spore appendages (strains E204, R28058, SSCC33587, and UMC4404) were treated with urea and beta-mercaptoethanol, which removed the exosporium from all spores but not the appendages for strains E204 and R28058 (see Fig. S1A in the supplemental material). This indicates that, at least in these two strains, appendages are chemically resistant to these conditions and that they may be anchored more tightly, perhaps to the inner spore. Although *C. sordellii* spores were harvested and prepared for imaging using gentle methods, it was common to observe spores across all strains that were devoid of all inner spore structures except the spore coat (Fig. S1B to D). The undercoat appears to fill the entire central region of these spores (Fig. S1D). In addition, these naturally occurring spore variants showed the normal appearance of appendages, suggesting that appendages may originate from the coat (Fig. S1B). Although possible, it is unlikely that these spore entities are remnants from a germinated spore as spores were produced in sporulation medium, which is not conducive to germination.

10.1128/mSphere.00343-17.1 FIG S1  TEM on spores of strain R28058 after urea and beta-mercaptoethanol treatment showing appendages attached to the inner spore (A). Naturally occurring spore variant missing part of inner spore in strain W2922 (B) and strain ATCC 9714 (C). Boxed area in panel C is enlarged in panel D. TEM performed on negatively stained whole spores (A and B) or spore sections (C and D). Ap, appendage; Ex, exosporium; IS, inner spore; OC, outer coat; IC, inner coat; UC, undercoat. OC, IC, and UC make up the spore coat. Bars, 0.5 µm (A to C) and 0.1 µm (D). Download FIG S1, JPG file, 2.8 MB. Copyright © 2017 Rabi et al. 2017 Rabi et al. This content is distributed under the terms of the Creative Commons Attribution 4.0 International license.

### Ultrastructure of *C. sordellii* spores.

**FIG 3:**
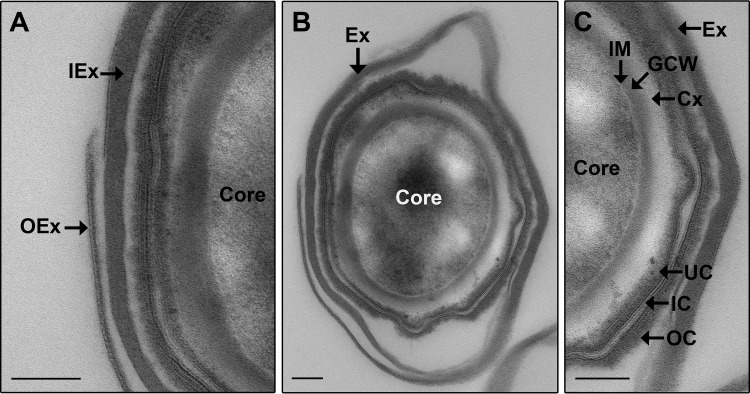
TEM on spore section of strain ATCC 9714. A transverse spore section is presented. Panel A is a magnified view of the left side of panel B. Panel C is a magnified view of the right side of panel B. Panel B shows the complete spore section. IEx, inner exosporial layer; OEx, outer exosporial layer; Ex, exosporium; IM, inner membrane; GCW, germ cell wall; Cx, cortex; OC, outer coat; IC, inner coat; UC, undercoat. A single layer each of IEx and OEx makes up the Ex. Bars, 0.1 µm.

Sections of spores were imaged by TEM to examine the internal structures (Fig. 3). The inner spore consists of the core, inner membrane (IM), germ cell wall (GCW), cortex (Cx), and coat. The coat is made up of three sections consisting of the electron-dense undercoat (UC) and outer coat (OC), both of which sandwich a striated inner coat (IC) (Fig. 3B and C). An outer membrane was not discernible in the spore sections, and this has also been noted in *B. subtilis*, *B. cereus*, *Bacillus sphaericus*, and *Clostridium novyi* spore sections (34–37). The exosporium surrounds the inner spore and consists of a thick inner layer (IEX) and a thin outer layer (OEX) (Fig. 3A and B) as observed in 100% of spores (at least 10 spores visualized by TEM cross section). The width of each exosporial layer was measured for 5 spores, and the average width was 37 nm for the thick inner layer (widths ranged from 31 nm to 48 nm) and 3 nm for the thin outer layer (widths ranged from 2 nm to 3 nm). Although most spores had the dilayered exosporium intact, Fig. 3A shows a spore with part of the outermost layer of the exosporium broken off and is included to clearly highlight the two layers.

### The *C. sordellii* exosporium wraps around the inner spore.

**FIG 4:**
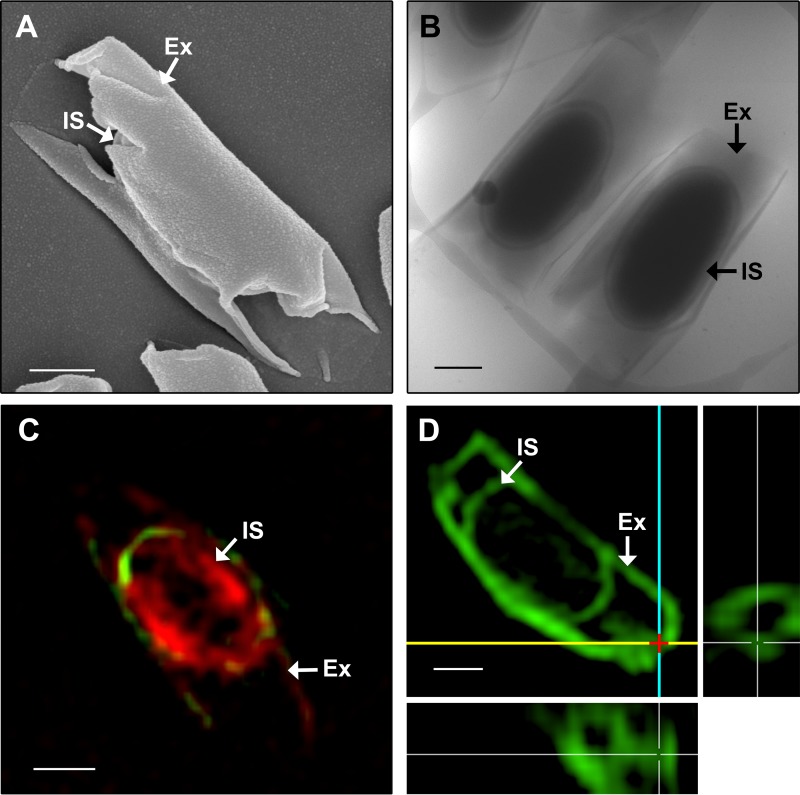
Spores of strain ATCC 9714 imaged by SEM (A), cryo-EM (B), and 3D-SIM (C and D) with spores labeled with lipid dyes DiO and FM 4-64FX (C) or DiO and spore-specific antibodies (D). The 3D-SIM image presented in panel D is a maximum-intensity projection of the *z* series through the spore volume (main image), supplemented by the *xz* projection (bottom panel) and *yz* projection (right panel). The red cross marks the distal end of one spore pole, the yellow line marks the *x* plane, and the blue line marks the *y* plane. The exosporial openings are visible in the *xz* and *yz* projections, where the spore has been sliced along the yellow line and is viewed from the top or where the spore has been sliced along the blue line and is viewed from the side of the spore, respectively. IS, inner spore; Ex, exosporium. Bar, 0.5 µm.

The imaging techniques used in the studies described above do not provide details of the spore surface. In addition, they require the spores to be dehydrated, which could result in a distorted presentation of the exosporium. For this reason, SEM was used to gain information on the spore topography (Fig. 4A) and cryo-EM and three-dimensional structured illumination microscopy (3D-SIM) were used to image spores in their hydrated state (Fig. 4B to D). The exosporium appears to sit close to the inner spore and fans out at the spore poles (Fig. 4). SEM showed that the exosporium is a smooth layer that wraps around an inner spore, with the exosporial poles appearing to be open (Fig. 4A). When analyzed by SEM, 9 out of 10 spores had an exosporium that was clearly open (data not shown). The one spore that did not have a clearly open exosporium appeared to have the open ends of the exosporium pinched together, and this may be the result of spore dehydration during SEM processing. 3D-SIM of DiO (3,3′-dioctadecyloxacarbocyanine perchlorate)- and FM 4-64FX [fixable analog of *N*-(3-triethylammoniumpropyl)-4-(6-(4-(diethylamino) phenyl) hexatrienyl) pyridinium dibromide]-stained spores, presented here in a single z-stack (Fig. 4C), indicated that the *C. sordellii* exosporium and inner spore are hydrophobic, as they stained with lipophilic dyes. The series of z-stacks, which allow the entire spore to be visualized, can be found in the supplemental material (Fig. S2). Imaging of unstained spores showed negligible autofluorescence in comparison to stained spores (data not shown). The sample preparation used for 3D-SIM retains spore hydration and supports the observation that the exosporium appears to be open at the spore poles, as an exosporial opening is clearly seen in this region (Fig. 4D). The exosporial opening in the spore presented in Fig. 4D measured 0.2 µm in diameter. In some clostridial species, such as *Clostridium pasteurianum*, the exosporium appears to be attached to the rest of the spore by suspensor-like structures (38, 39). No such suspensors were observed in the *C. sordellii* spore. Instead, a defined interspace exists between the entire inner spore and the exosporium (Fig. 4B).

10.1128/mSphere.00343-17.2 FIG S2  Spore of strain ATCC 9714 labeled with DiO and FM 4-64FX, imaged by 3D-SIM, and presented as a series of z-stacks. Bar, 1 µm. Download FIG S2, JPG file, 0.6 MB. Copyright © 2017 Rabi et al. 2017 Rabi et al. This content is distributed under the terms of the Creative Commons Attribution 4.0 International license.

### *C. difficile* spore morphology and ultrastructure.

**FIG 5:**
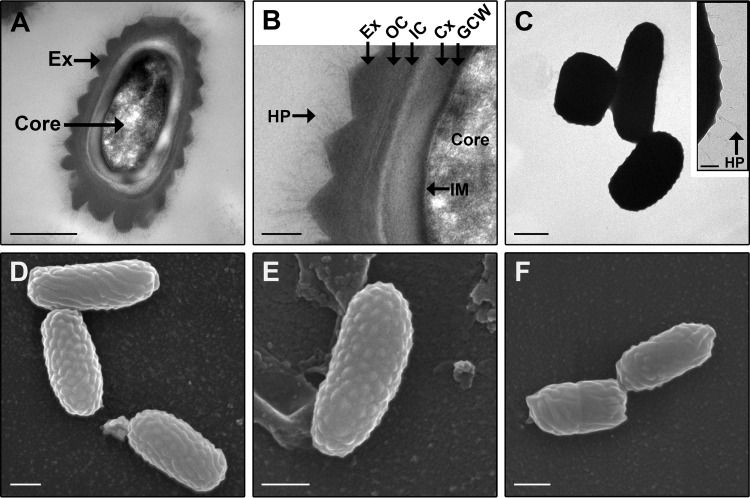
Spores of *C. difficile* imaged by TEM and SEM. (A and B) TEM on spore section of strain CD196 (A) with an area of the image magnified (B). (C) TEM on negatively stained whole spores of strain M7404 with the inset showing a magnified view of the spore edge. (D to F) SEM on spores of strains CD196 (D), 630E (E), and CD47 (F). Ex, exosporium; HP, hair-like projections; IM, inner membrane; GCW, germ cell wall; Cx, cortex; OC, outer coat; IC, inner coat. Bars, 0.5 µm (A and C to F) and 0.1 µm (B and C inset).

*C. sordellii* and *C. difficile* show 77% sequence similarity in their 16S rRNA genes (1) and are therefore phylogenetically closely related. Consequently, the *C. difficile* spore was imaged using TEM and SEM to enable a direct comparison of the spores to those of *C. sordellii* (Fig. 5). The *C. difficile* strains used in this study have not been previously imaged. Furthermore, TEM imaging of whole *C. difficile* spores has never been performed, and SEM imaging of *C. difficile* spores has been limited and often lacking in providing structural details of the spores (40–43). The imaging showed that as in *C. sordellii*, the *C. difficile* spore has an inner spore consisting of the core, inner membrane (IM), germ cell wall (GCW), cortex (Cx), and coat (Fig. 5A and B). The coat in *C. difficile* appears to be comprised of two layers (Fig. 5B) consisting of the inner coat (IC) and the more electron-dense outer coat (OC). Both the inner coat and the outer coat appear to be striated. Unlike the *C. sordellii* spore, an electron-dense exosporial layer (Ex) surrounds the inner spore and is covered with hair-like projections (HP) (Fig. 5A to C). TEM on spore sections (Fig. 5A and B) and TEM (Fig. 5C) and SEM (Fig. 5D to F) on whole spores showed that the exosporium of *C. difficile* is bumpy in all the strains examined. In addition, unlike the spores of *C. sordellii*, the exosporium completely encloses the inner spore and no gap is visible between the exosporium and inner spore (Fig. 5A to C).

## DISCUSSION

Despite the importance of *C. sordellii* in disease, its spores, which are critical to the infection process, have not been well characterized. The imaging studies presented here have provided new insights into the morphology of this important cell type. TEM was used to compare the spores of 16 different *C. sordellii* strains. Measurements of the inner spore length and width, exosporial length, and presence and length of appendages have revealed some differences in spore dimensions between strains (Fig. 1 and 2). However, these differences did not correlate with any other strain characteristics, including clade, geographical location, host origin, or virulence. This is in keeping with a previous study on *C. sordellii* spores that showed no correlation between the presence of spore appendages from particular strains and strain characteristics such as toxicity, motility, or the ability to digest meat (17). The lack of any correlation between spore dimensions and strain characteristics, such as strain niche and virulence, has also been noted in *Bacillus* species (19, 44). Furthermore, within a single strain, there were variations in the dimensions between spores, which was seen most strikingly in inner spore length, exosporial length, and appendage length (Fig. 2A, C, and D), an observation that is intriguing given the tight regulatory processes controlling sporulation (27). In *B. cereus*, it was shown that spores with a shorter exosporium had a greater ability to remain adhered to stainless steel after standard cleaning procedures than did spores with a longer exosporium (26). Perhaps, having a range of spore sizes or variants produced from a strain is advantageous to *C. sordellii* and other bacterial species (19). In the case of adherence, for example, short spore variants might adhere strongly, ensuring retention within that site, while long spore variants, which adhere more weakly, may allow for wider dissemination.

Two exosporial layers are present in the *C. sordellii* spore (Fig. 2). A multilayered exosporium has been noted previously in spores of other species such as *B. anthracis* and *Clostridium botulinum* (45, 46); however, the functional significance of these features is unknown. When viewed under high magnification, the *C. sordellii* exosporium appears to form a hexagonal array (Fig. 1H), which is also seen with the exosporium from *B. cereus*, *B. anthracis*, *B. thuringiensis*, *C. botulinum*, and *C. pasteurianum* spores (23, 24, 38, 45–47). In *B. cereus*, the hexagonal units measured approximately 8 nm in diameter (23). These hexagonal subunits are believed to form pores within the exosporium that are large enough to allow the passage of small molecules, such as germinants, but small enough to prevent the entry of degradative enzymes and antibodies through the exosporium to the underlying spore (23, 24, 38, 45–47). In this context, it is puzzling that the *C. sordellii* exosporium has large openings at its poles (Fig. 4). The literature suggests that, unlike spores of *Bacillus* species, where the baggy exosporium appears to completely surround the inner spore, clostridial spores that possess a baggy exosporium have a large opening in the exosporium at either one or both ends of this structure (38, 39, 48–50). Thus, the notion that the exosporium acts as a molecular sieve in clostridial spores may not be correct in this species. The function of an open exosporium is unclear; however, in *Clostridium sporogenes*, newly formed vegetative bacteria were shown to emerge through these openings upon germination (50). The open exosporium may also allow the area between the inner spore and exosporium, known as the interspace, to be penetrated by environmental material. Perhaps, this enhances the ability of the inner spore to sense and respond to the local environment. In support of this hypothesis, electron microscopy images of sporulating *C. botulinum* cells show that the exosporium sits tightly against the inner spore within the mother cell but that the exosporium inflates when the mature spore is released and environmental fluid enters the interspace region (18).

Morphologically, the smooth, baggy, balloon-like *C. sordellii* exosporium has a very different appearance from that of *C. difficile*, in which the exosporium is bumpy, has hair-like projections, and sits tightly and completely around the inner spore (Fig. 5) (33). It should be noted that spores of some *C. difficile* strains have a smooth exosporium without the hair-like projections; however, the exosporium still tightly surrounds the inner spore (33). To our knowledge, there are no other spores studied to date that have the same exosporial morphology as that of *C. difficile*. It has been suggested that spores of *B. subtilis* have an exosporial layer which also sits tightly against the inner spore (51). However, this layer is thin, lacks distinctive bumps, may not completely cover the inner spore, and is possibly not a true exosporium but rather another layer of the spore coat (19, 30, 51). The hair-like projections on the surface of *C. difficile* spores (Fig. 5A to C) (33) are a feature also observed in spores of *B. anthracis*, *B. cereus*, and *B. thuringiensis*. Unlike *C. difficile*, all the spores of *Bacillus* species with hair-like projections have a baggy exosporium (19), similar to the exosporium of *C. sordellii*. However, hair-like projections were not observed on spores of any *C. sordellii* strain studied in this or previous works (Fig. 1 and 3) (17, 18). In addition, tubular appendages observed in *C. sordellii* spores can be present in spores of *Bacillus* species; however, these have not been observed in *C. difficile* spores (Fig. 1 and 5) (19, 33, 52).

This study presents for the first time a both broad and in-depth analysis of *C. sordellii* spore structures and compares these structures to a closely related bacterium, *C. difficile*. A range of microscopy techniques used in this analysis has provided a new understanding of the morphology of these important cell types, with particular emphasis given to the exosporium. All *C. sordellii* spores examined had an inner spore surrounded by a balloon-like exosporium. However, spores of different strains varied somewhat from one another in the length of the exosporium, the length and width of the inner spore, and the presence and length of appendages. The *C. sordellii* exosporium which appeared to be open may be a characteristic that is specific to spores of clostridial species with a balloon-like exosporium. This study has expanded our knowledge of *C. sordellii* spore morphology and has highlighted the differences and similarities between spores from different bacterial species and genera.

## MATERIALS AND METHODS

### Bacterial strains.

The *C. sordellii* strains used in this study and their characteristics are listed in Table S1 in the supplemental material. The *C. difficile* strains used in this study were CD196, M7404, 630E, and CD47 (53, 54). *C. sordellii* and *C. difficile* were grown at 37°C in a Coy anaerobic chamber in 3.7% brain heart infusion containing 0.5% yeast extract (BHIS) broth or agar plates supplemented with 0.1% l-cysteine–HCl and 0.05% glucose.

10.1128/mSphere.00343-17.3 TABLE S1  *C. sordellii* strains used in this study. Source for ATCC 9714, supplemental references 1 and 2; all other strains, supplemental reference 1. Download TABLE S1, PDF file, 0.1 MB. Copyright © 2017 Rabi et al. 2017 Rabi et al. This content is distributed under the terms of the Creative Commons Attribution 4.0 International license.

### Spore production, spore purification, and exosporial removal.

*C. sordellii* spores were prepared by plating overnight cultures onto 3% Trypticase soy-2% yeast extract (TY) agar plates supplemented with 0.1% sodium thioglycolate and incubating them for 10 days at 37°C in a Coy anaerobic chamber. Spores were harvested by centrifugation and washed once with distilled water at 8,873 × *g* followed by at least two washes at 4,194 × *g*. After each centrifugation, cellular debris was removed from the top of the spore pellet and spores were gently resuspended in sterile distilled water. *C. difficile* spores were prepared by the same method used for *C. sordellii* spores except that overnight cultures were inoculated into TY broth supplemented with 0.1% sodium thioglycolate instead of TY agar. Spore stocks were stored in distilled water at 4°C. The exosporium was removed from spores as per the preparation of spore extracts in *B. anthracis* using 8 M urea and 2% beta-mercaptoethanol (55).

### Electron microscopy.

All electron microscopy was performed at the Ramaciotti Centre for Cryo-Electron Microscopy, Monash University, Australia.

### TEM.

Whole spores were adsorbed onto Formvar carbon-coated 400-mesh grids (Micromon, Clayton, Australia) and negatively stained with 2% uranyl acetate. Grids were imaged with a Hitachi H7500 electron microscope at 80-kV accelerated voltage. Spore measurements were performed from these images using Fiji software (56). For each strain, 20 spores each from 2 biological replicates were measured for their inner spore and exosporium sizes. For each strain, the number of appendages present on 30 spores was measured for appendage lengths. To determine spore internal structures, spores were fixed with 2.5% glutaraldehyde in 0.1 M cacodylate buffer for 1 h at ambient temperature followed by 12 h at 4°C. The samples were postfixed in 1% osmium tetroxide in MilliQ water for 1 h. Samples were rinsed in cacodylate buffer and progressively dehydrated once in 50%, 70%, and 90% and twice in 100% and absolute ethanol for 10 min each. Samples were placed in propylene oxide twice for 10 min each and then placed in 25% resin in propylene oxide for 1 h, 50% resin in propylene oxide overnight, and 100% resin three times for 2 h each. Samples were then left in resin and allowed to polymerize at 70°C. Thin sections were cut using an ultramicrotome (Leica Ultracut UCT) and collected on copper-palladium 150-mesh grids (ProSciTech, Brisbane, Australia). Sections were poststained with 2% uranyl acetate and lead citrate. Grids were observed using the FEI Tecnai T12 microscope at 120-kV accelerated voltage. All steps were performed at ambient temperature unless otherwise noted.

### SEM.

Spores were adsorbed onto glass coverslips and processed as for spore sections used in TEM with the following exceptions: following sample dehydration in absolute ethanol, samples were placed in the following ratios of ethanol to hexamethyldisilazane for 10 min each: 2:1, 1:1, and 1:2. They were then placed twice in hexamethyldisilazane alone and allowed to dry. The glass coverslips containing the spores were mounted onto aluminum stubs and sputter coated with gold. Samples were imaged using either the Hitachi S570 or FEI Nova nano-SEM 450 at 15-kV accelerated voltage.

### Cryo-EM.

Spores were fixed in 4% paraformaldehyde in 0.1 M cacodylate buffer for 2 h at ambient temperature and then stored in 1% paraformaldehyde in 0.1 M cacodylate buffer at 4°C. Spores were applied to lacy carbon grids (ProSciTech) and processed as per the preparation of frozen hydrated samples in reference 46. Samples were imaged using the FEI Tecnai T12 microscope at 120-kV accelerated voltage, with images collected on a 4K FEI Eagle camera.

### 3D-SIM.

*C. sordellii* strain ATCC 9714 spores were prepared as follows: spores labeled with DiO and FM 4-64FX (Invitrogen, Thermo Fisher, CA, USA) were incubated with DiO for 3 h at 37°C and then FM 4-64FX overnight, followed by fixation in 4% paraformaldehyde for 4 h. For spores labeled with antibody and DiO, the spores were blocked in 2% bovine serum albumin for 1 h, labeled overnight with antibodies raised in rabbits against strain ATCC 9714 whole spores (SAHMRI, Adelaide, Australia), and then incubated with an anti-rabbit Alexa Fluor 488-conjugated secondary antibody (Invitrogen, Thermo Fisher, CA, USA) for 1 h to allow visualization. Spores were then incubated with DiO for 3 h at 37°C, followed by fixation in 4% paraformaldehyde for 4 h. Spore samples were spotted and air dried onto microscope slides (Menzel), mounted in ProLong Gold antifade reagent (Invitrogen, Thermo Fisher, CA, USA), coverslipped (no. 1.5 thickness; Zeiss), and sealed with nail polish. Spores were imaged using a DeltaVision OMX V3 three-dimensional structured illumination microscopy (3D-SIM) imaging system fitted with Blaze technology (GE Healthcare, Issaquah, WA) (57). Images were acquired and processed as previously described (58). Images were analyzed using IMARIS software (Bitplane Scientific, Oxford Instruments, Switzerland). All steps were carried out at ambient temperature unless otherwise stated.
