# Supplementary figures and images for: Structural Characterization of Clostridium sordellii Spores of Diverse Human, Animal, and Environmental Origin and Comparison to Clostridium difficile Spores
Source: mSphere. 2017 Oct 4;2(5):e00343-17. doi: 10.1128/mSphere.00343-17 (PMC5628289; doi:10.1128/mSphere.00343-17)

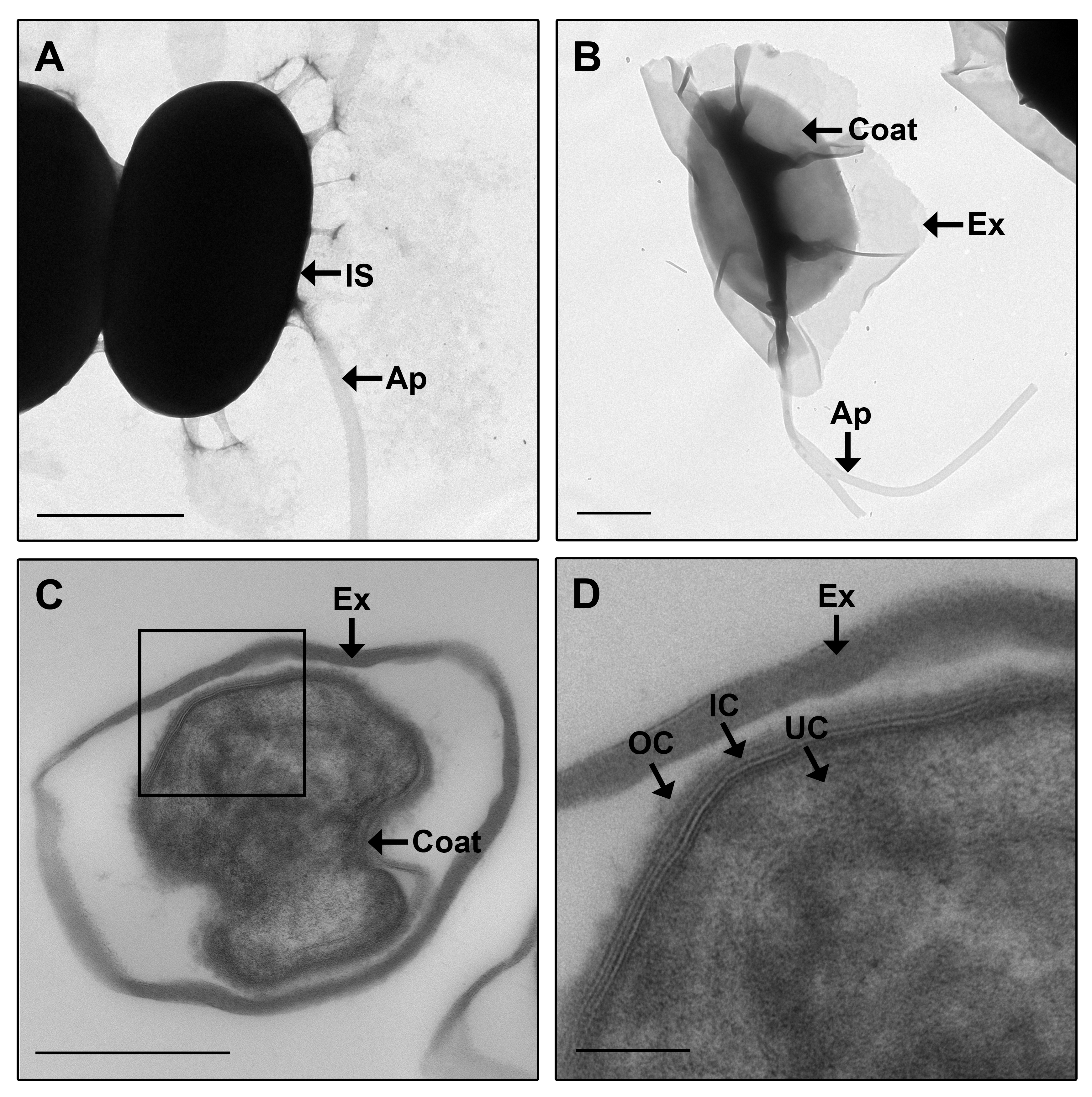

Supplement: FIG S1 [file sph005172376sf2.jpg]

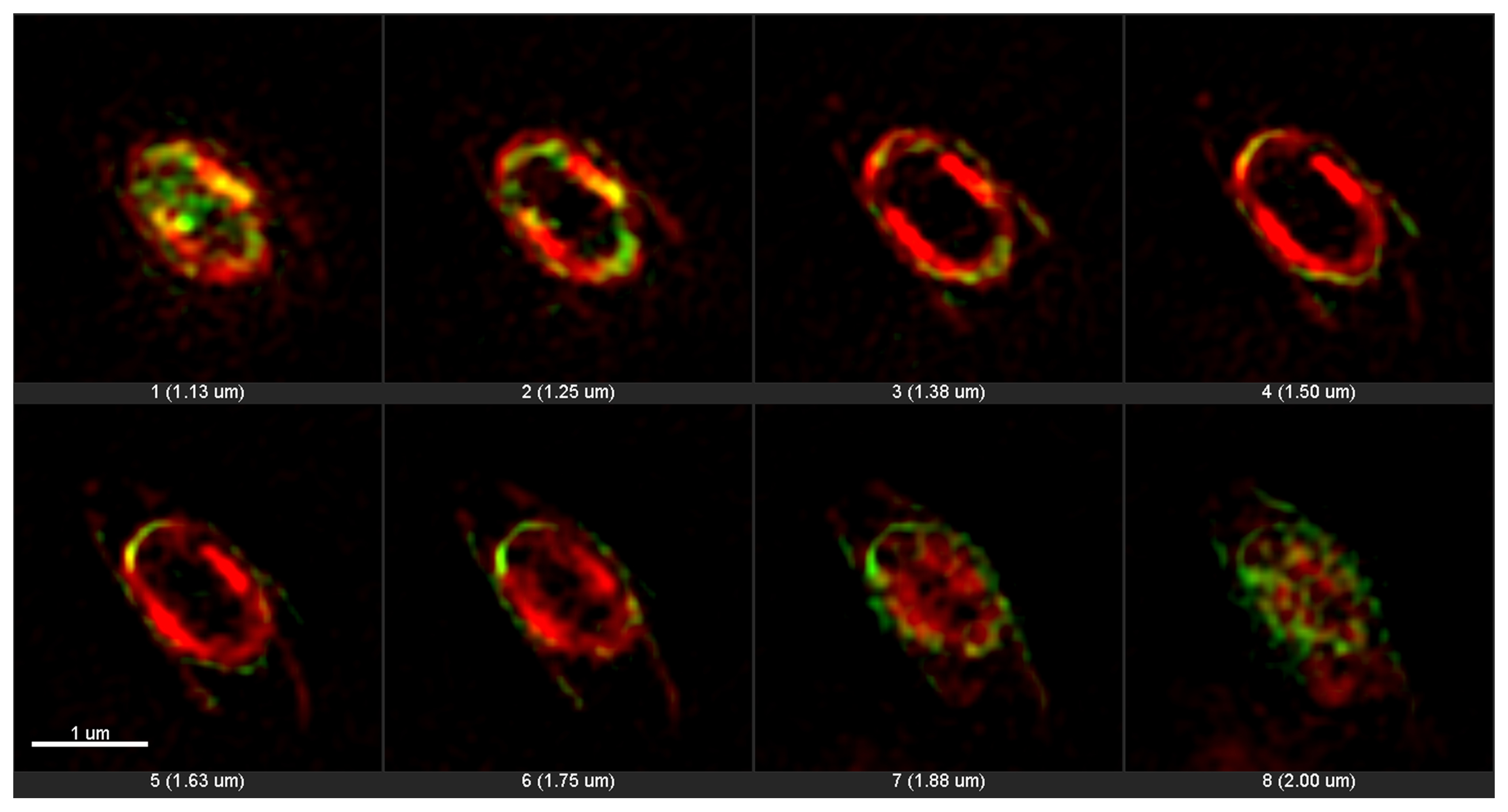

Supplement: FIG S2 [file sph005172376sf3.jpg]
