# Supplementary material for: Structural Characterization of Clostridium sordellii Spores of Diverse Human, Animal, and Environmental Origin and Comparison to Clostridium difficile Spores
Source: mSphere. 2017 Oct 4;2(5):e00343-17. doi: 10.1128/mSphere.00343-17 (PMC5628289; doi:10.1128/mSphere.00343-17)
Supplement: TABLE S1 [file sph005172376st1.pdf]

Supplementary Table 1

Strain	Host origin	Geographic origin	Major toxin genes	Clade
ATCC9714	Human<br>Oedema	South America	$tcsL^{+}$ / $tcsH^{-}$	1
DA108	Human<br>Post-partum endometritis	USA	$tcsL^{-}$ / $tcsH^{-}$	3
UMC2	Human<br>Allograft isolate	USA	$tcsL^{-}$ / $tcsH^{-}$	3
UMC4401	Human<br>Allograft isolate	USA	$tcsL^{-}$ / $tcsH^{-}$	2
UMC4404	Human<br>Allograft isolate	USA	$tcsL^{-}$ / $tcsH^{-}$	3
JGS6364	Bovine<br>Myonecrosis	USA	$tcsL^{+}$ / $tcsH^{-}$	1
JGS6382	Bovine<br>myonecrosis	USA	$tcsL^{+}$ / $tcsH^{+}$	NA*
SSCC26591	Human<br>Blood isolate	Australia	$tcsL^{-}$ / $tcsH^{-}$	2
SSCC33587	Human<br>Blood isolate	Australia	$tcsL^{-}$ / $tcsH^{-}$	3
SSCC35109	Human<br>Blood isolate	Australia	$tcsL^{-}$ / $tcsH^{-}$	4
SSCC42239	Human<br>Blood isolate	Australia	$tcsL^{-}$ / $tcsH^{-}$	2

E204	Human clinical	Australia	*tcsL*$^-$ / *tcsH*$^-$	3
R15892	Human clinical	UK	*tcsL*$^-$ / *tcsH*$^-$	1
R28058	Human<br><br>Crushed hand	UK	*tcsL*$^-$ / *tcsH*$^-$	4
W2922	Veterinary isolate	UK	*tcsL*$^-$ / *tcsH*$^-$	2
W3026	Veterinary isolate	UK	*tcsL*$^-$ / *tcsH*$^-$	2

*Does not fit into a defined clade

Table S1. *C. sordellii* strains used in this study. Source for ATCC9714 (1, 2), all other strains (1).

1. **Couchman EC, Browne HP, Dunn M, Lawley TD, Songer JG, Hall V, Petrovska L, Vidor C, Awad M, Lyras D, Fairweather NF.** 2015. *Clostridium sordellii* genome analysis reveals plasmid localized toxin genes encoded within pathogenicity loci. BMC Genomics. **16**(1)**:**doi:10.1186/s12864-015-1613-2.
2. **Hall IC, Scott JP.** 1927. *Bacillus Sordellii*, a Cause of Malignant Edema in Man. The Journal of Infectious Diseases **41:**329-335.
